# Network Analysis of Functional Brain Connectivity Driven by Gamma-Band Auditory Steady-State Response in Auditory Hallucinations

**DOI:** 10.1007/s40846-015-0004-0

**Published:** 2015-02-06

**Authors:** Jun Ying, Dan Zhou, Ke Lin, Xiaorong Gao

**Affiliations:** 1 Department of Biomedical Engineering, PLA General Hospital, Beijing, 100853 People’s Republic of China; 2 Department of Biomedical Engineering, School of Medicine, Tsinghua University, Beijing, 100084 People’s Republic of China

**Keywords:** Functional connectivity, Auditory steady-state response (ASSR), Directed transfer function (DTF), Graph theory, Auditory hallucinations

## Abstract

The auditory steady-state response (ASSR) may reflect activity from different regions of the brain. Particularly, it was reported that the gamma-band ASSR plays an important role in working memory, speech understanding, and recognition. Traditionally, the ASSR has been determined by power spectral density analysis, which cannot detect the exact overall distributed properties of the ASSR. Functional network analysis has recently been applied in electroencephalography studies. Previous studies on resting or working state found a small-world organization of the brain network. Some researchers have studied dysfunctional networks caused by diseases. The present study investigates the brain connection networks of schizophrenia patients with auditory hallucinations during an ASSR task. A directed transfer function is utilized to estimate the brain connectivity patterns. Moreover, the structures of brain networks are analyzed by converting the connectivity matrices into graphs. It is found that for normal subjects, network connections are mainly distributed at the central and frontal–temporal regions. This indicates that the central regions act as transmission hubs of information under ASSR stimulation. For patients, network connections seem unordered. The finding that the path length was larger in patients compared to that in normal subjects under most thresholds provides insight into the structures of connectivity patterns. The results suggest that there are more synchronous oscillations that cover a long distance on the cortex but a less efficient network for patients with auditory hallucinations.

## Introduction

An abnormal auditory steady-state response (ASSR) has been found in electroencephalography (EEG) and magnetoencephalography (MEG) signals of schizophrenia (SZ) patients [1]. The ASSR is an oscillatory brain response classically evoked by click trains at a constant and rapid rate or by amplitude-modulated (AM) tones [2]. In the clinic, the ASSR has extensive application, such as for testing hearing sensitivity and as a marker of conscious state during anesthesia. Initial studies suggested that oscillations in the gamma band of an electroencephalogram can reflect the local and global communications of the brain [3]. SZ is a mental disorder characterized by auditory hallucinations, impaired consciousness, breakdown of thought processes, and poor emotional responsiveness. Studies performed on SZ patients have found that the gamma-band ASSR is closely associated with perception and cognition. A difference of the gamma-band ASSR between healthy persons and patients with SZ has been observed. Mulert et al. found that compared to healthy controls, the power spectral density of the 40-Hz ASSR is significantly lower in SZ patients [4]. Spencer et al. found a decrease in interhemispheric phase locking in the ASSR from the primary auditory cortices of SZ patients [5].

The research mentioned above implies that instead of a simple response to stimulus, the ASSR can indicate the global information transmission process between different brain regions in a more complex pattern than that obtained using spontaneous EEG. Auditory hallucination is one of the common symptoms of SZ. The gamma-band ASSR is supposed to be a usefull probe for detecting auditory neurological dysfunction. The traditional ASSR detection technique based on power spectral density analysis (PSDA) cannot accurately detect the widely distributed properties of the ASSR and the relation between distinct regions. Yan et al. introduced functional connectivity into steady-state visual evoked potential (SSVEP) analysis and proved that directed transfer function (DTF) was useful for observing the common patterns of distinct channels [6]. SSVEP and the ASSR are steady-state evoked potentials (SSEPs). Generally, the principle of all SSEPs is somewhat similar to that of SSVEP. Although functional connectivity has been found useful for SSVEP, it has not been commonly applied to evoked ASSR.

An increasing number of researchers have realized that conceptualizing brain networks is helpful to understand the organized behavior of cortical regions [7, 8]. Functional connectivity in brain networks indicates the activity correlation between different nerve cell clusters. Various methods, such as structural equation modeling and partial directed coherence, have been proposed for estimating brain connectivity [9, 10]. Kaminski et al. proposed a multivariate spectral method of the DTF for evaluating the simultaneous processes in all channels to estimate the flow activity between different channels with a given direction as a function of frequency [11]. The DTF is based on the theory of Granger causality and builds a multivariate autoregressive (MVAR) model of all EEG channel recordings [11, 12]. Several works have used the DTF to estimate brain functional connectivity and obtained accurate patterns [5, 13, 14].

The present study investigates the central nervous mechanism of auditory hallucinations using the ASSR. Brain functional analysis is applied to evoked potential signal processing. The brain functional connectivity pattern of the ASSR is estimated. Based on this pattern, the network structures and neural mechanisms of patients with auditory hallucinations are examined. The connectivity matrices obtained using the DTF are converted into a brain functional connectivity graph with the position information of each channel. With the graph, some concepts and tools from graph theory are used to explore the characteristics of the network. The structures of connectivity patterns of SZ patients with hallucination are studied by computing the cluster coefficient (*Cp*) and path length (*Lp*).

## Methods

### Subjects

Ten healthy subjects (3 females, 7 males, mean age: 27 years, standard deviation: 3.2 years, range: 20–40 years) and 5 SZ patients suffering from auditory hallucinations for more than a year (1 female, 4 males, mean age: 35 years, standard deviation: 4.7 years, range: 20–45 years) participated in the experiments. All subjects were right-handed and had normal hearing. Participants were required to be seated in a shielded quiet room in order to acquire good-quality EEG data. Informed consent was received from every subject before the experiments according to the Declaration of Helsinki.

### EEG Recording

**Fig. 1:**
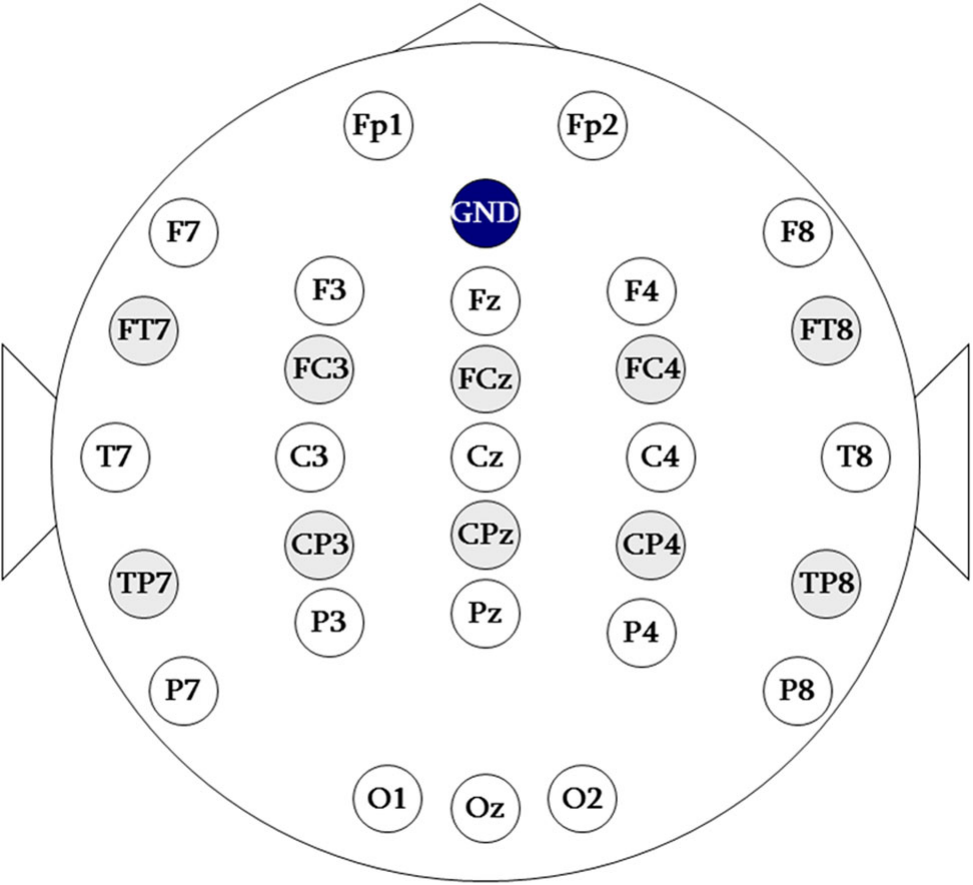
Positions of 30 channels used for EEG acquisition

A Synamps2 (NeuroScan Inc.) system was used to record multichannel EEG signals. In the experiment, 30 channels placed according to the international 10–20 electrode system were used (Fig. 1). The reference electrode was placed on the nose. The impedance of each channel was kept below 10 kΩ. Data were recorded at a sampling rate of 1,000 Hz and then bandpass-filtered at 0.05 and 200 Hz. The set-up above was fixed for all experiments in this study.

### Experimental Paradigm

In order to acquire a better ASSR, instead of click trains, a repeated chirp at a rate of 40 Hz was utilized in the experiments. Stimuli were delivered binaurally through earphones at an intensity of 60 dB. The sounds were played for 50 s. The silent inter-stimulus interval (ISI) was 5 s. The chirp signals were created based on the cochlear traveling wave theory proposed by Don et al. [15]. The sounds were produced by a digital signal processor (DSP) TMS320C5515 (TI Inc.). The sounds had been tested and found to be precise enough to allow one to control for error within 5 μs. ER2 insert earphones (Etymotic Research) were selected because they can effectively avoid electromagnetic interference.

### Functional Connectivity Estimation

Let *t* refer to time, *N* refer to channel number, and *x*_*i*_(*t*) refer to the EEG data of channel *i*. Then, multichannel EEG waveforms *X* can be represented as:

1 $$X = \left[ {x_{1} (t),\,x_{2} (t), \ldots ,\,x_{N} (t)} \right]^{T}$$

The resulting data *X* are fit to an MVAR model [16]. Based on the fit MVAR model, the data epoch properties are described in the following form:

2 $$\sum\limits_{k = 0}^{p} \varLambda \left( k \right)X\left( {t - k} \right) = E\left( t \right);\quad \varLambda \left( 0 \right) = I$$

where Λ(*k*), an N × N matrix, is the MVAR model parameters and *E*(*t*) is an uncorrelated white noise vector with multivariate zero mean. The parameter *p* refers to the order of the MVAR model and affects modeling performance. The spectral properties of the investigated process are determined by transforming Eq. (2) into the frequency domain:

3 $$X(f) = \varLambda^{ - 1} (f)E(f) = H(f)E(f)$$

where,

4 $$\varLambda (f) = \sum\limits_{k = 0}^{p} {\varLambda (k)e^{ - j2\pi f\Delta tk} }$$

In Eq. (3), the MVAR model is used as a black box. The noise is the input and the signal can be obtained from the output of the transfer matrix of the model. In the system, *H*(*f*) is the transfer matrix of the system with elements *H*_ij_. All the information about the spectral properties and the interrelation between channels is contained in *H*(*f*). *H*_*ij*_ *(f)* is the elements in the transfer matrix referring to the directional function connection between output *i* and input *j* [11]. The DTF can be constructed from the transfer matrix [17]. The DTF is defined as:

5 $$\theta_{ij}^{2} (f) = \left| {H_{ij} (f)} \right|^{2}$$

where *θ*_*ij*_^*2*^ *(f)* is the non-normalized DTF. In order to compare the results with different power spectra, the results are normalization method by dividing each element in the DTF by the squared sums of all elements in the relevant row:

6 $$\gamma_{ij}^{2} (f) = \frac{{\left| {H_{ij} (f)} \right|^{2} }}{{\sum\limits_{m = 1}^{N} {\left| {H_{im} (f)} \right|^{2} } }}$$

A significance test is conducted to remove connections due to chance. In this study, computation of the DTF matrices was conducted using the eConnectome Toolbox in MATLAB (The MathWorks Inc.) [18]. The surrogate data method was used to select functional links not due to chance [17]. The number of shuffles for the surrogate data method was set to 1,000 and the statistical significance level was set to 0.01 %.

### Computation of Cluster Coefficient and Path Length

The complex brain functional network can be represented as a brain functional connectivity graph. Tools from graph theory are thus commonly used to represent exact mathematical computations of complex networks. In order to derive topological features, a threshold *T* is used to filter the DTF values in the connection matrix. Only DTF values that exceed *T* are considered. The most powerful connections are thus obtained. In this study, *T* values were selected in the range of 0–1 in increments of 0.001.

The cluster coefficient *Cp*, known as the transitivity, indicates the tendency for some network elements to form a local cluster. It is a measure of the number of edges that exist between nearest neighbors.

The variable *a*_*ij*_ is established to represent a potential link between nodes *i* and *j*. When a link exists, *a*_*ij*_ is equal to 1; otherwise it is 0. The local cluster coefficient *c*_*i*_ of node *i* expresses how likely *a*_*jm*_ = 1 for two neighbors *j* and *m* of node *i*. It can be obtained by counting the actual number of edges (*e*_*i*_) in the subgroup (*G*_*i*_) containing the neighbors of *i*. In *G*_*i*_, the maximum possible number of edges is *k*_*i*_(*k*_*i*_ − 1)/2, where *k*_*i*_ (the degree of node *i*) is the number of edges incident with the node. The local cluster coefficient is defined as the ratio between *e*_*i*_ and *k*_*i*_(*k*_*i *_− 1)/2 as follows:

7 $$c_{i} = \frac{{2e_{i} }}{{k_{i} (k_{i} - 1)}} = \frac{{\sum\nolimits_{j,m} {a_{ij} a_{jm} a_{mi} } }}{{k_{i} (k_{i} - 1)}}$$

The cluster coefficient *Cp* of the whole graph is represented by the average of *c*_*i*_ over all nodes *N*:

8 $$Cp = \frac{1}{N}\sum\limits_{i \in N} {c_{i} }$$

The path length, *Lp*, represents the average shortest length of a path connecting any two nodes in the graph. The number of connecting edges denotes the actual path length of a node. The path length of a graph is defined as the mean of the shortest geodesic lengths of all couples of nodes:

9 $$Lp = \frac{1}{N(N - 1)}\sum\limits_{i,j \in N,i \ne j} {d_{ij} }$$

where *d*_*ij*_ is the shortest length of the connection from node *i* to *j*. *Lp* indicates how well the elements of a graph are interconnected.

## Results

**Fig. 2:**
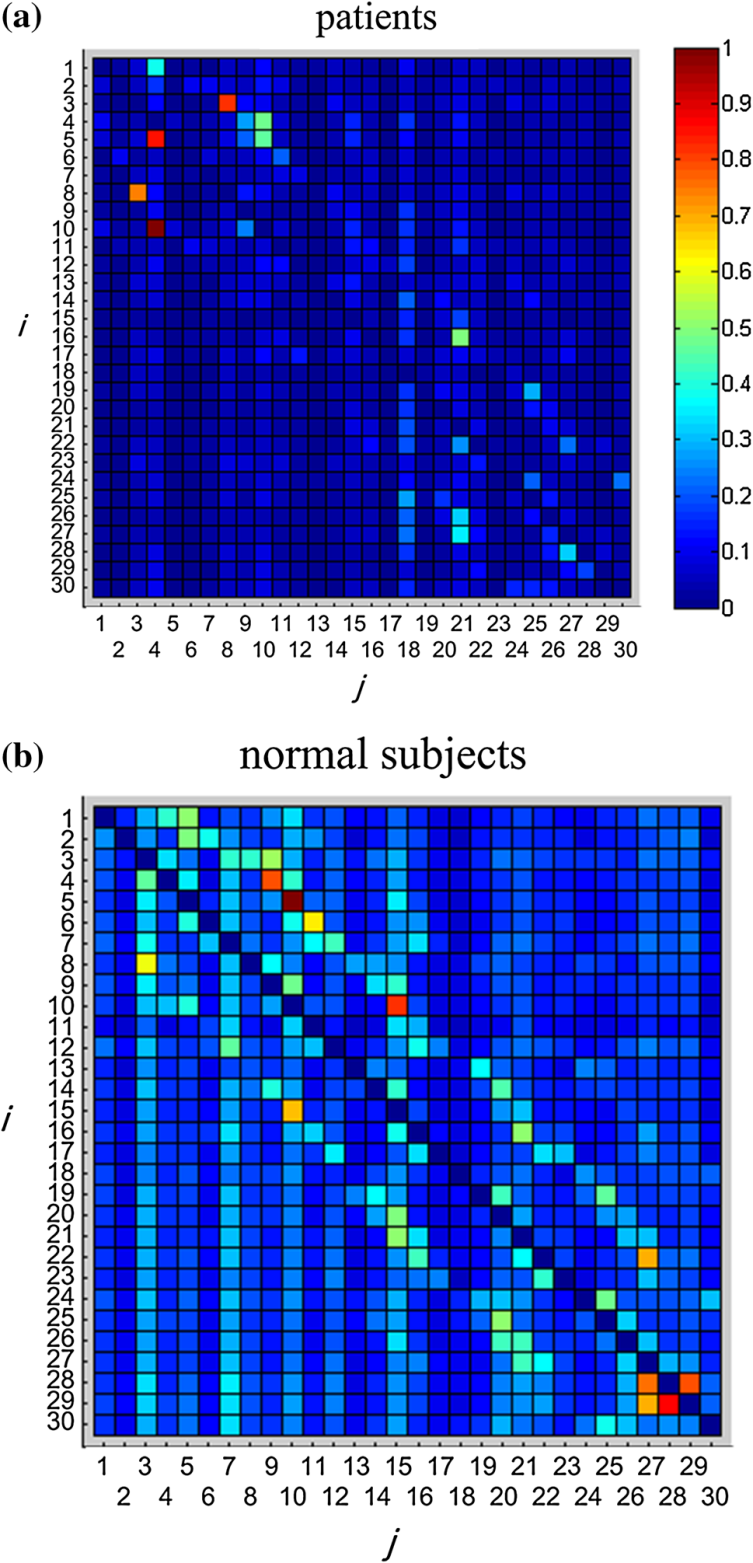
DTF matrices for **a** SZ patients and **b** normal subjects. The *color* of the *squares* indicates the connection strength between *i* and *j*. *DTF matrix* of normal subjects is lighter than that of SZ patients, indicating that normal subjects have more powerful brain functional connections than those of SZ patients

In an DTF matrix, each element indicates a correlation from node *i* to *j*. Nodes that have a stronger correlation have a greater connection. The DTF matrices for the two groups (10 normal subjects and 5 SZ patients, respectively) are plotted in Fig. 2. The average value of each matrix is represented by a different color. Light (dark) squares represent a strong (weak) directional function connection. The results show that the normal subjects have more powerful connections than do the SZ patients with auditory hallucinations, indicating that brain dysfunction can cause changes in brain functional connectivity.

**Fig. 3:**
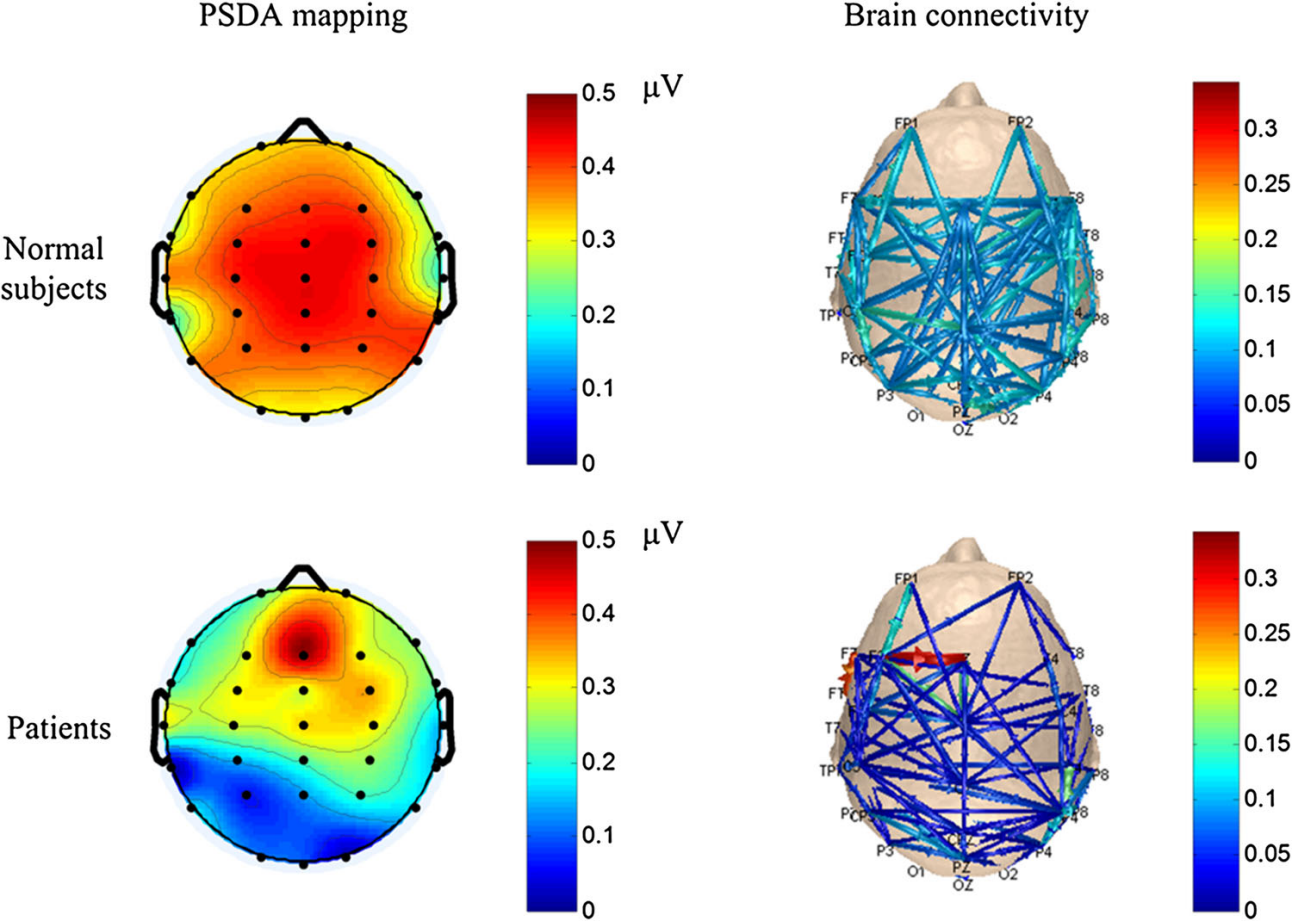
Average PSDA mappings and brain connectivity patterns. For the connectivity patterns, each *edge* of a connection is represented by *curves* *with arrows* that link one channel (source node) to another (sink node). The *color and size* of the curves indicate the strength normalization level of functional connectivity between channels

The functional connectivity patterns of all subjects are plotted in Fig. 3. They were obtained with the channel positions of the international 10–20 electrode system. The brain topographic mappings obtained by PSDA were calculated and plotted using the *topoplot* function of EEGLAB [19]. For the normal subjects, the distribution was wide, instead of being concentrated in the central area, as shown in the first row of Fig. 3. The maximum ASSR response to the 40-Hz stimulus mainly occurred at the frontal–central and parietal–occipital regions, with a symmetrical distribution. These results are consistent with the findings of Herdman [20]. The connections in the normal subjects between different regions are shown. The central regions are clearly visible. Furthermore, the sink regions, which receive information from other brain areas, are mainly distributed at the frontal–temporal regions. The second row shows the results for the SZ patients, which are significantly different from those for the normal subjects. The PSDA mapping of the patients is less widely distributed. The maximum ASSR response to the 40-Hz stimulus mainly occurred at the location of Fz in the front of the central region. The brain connectivity of patients shows an unordered pattern. There are strong connections between any two of the locations of FT3, Fz, and Cz.

**Fig. 4:**
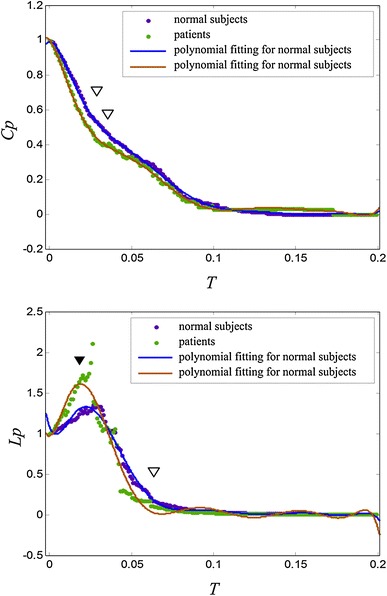
**a** *Cp* and **b** *Lp* values obtained for normal subjects and SZ patients. *Filled dots* indicate the original value and *solid lines* denote the polynomial fitting results (*inverted filled triangle* represents the statistically significant difference (*p* < 0.05); *inverted open triangle* represents the no statistically significant difference)

In order to investigate the difference between the normal subjects and the SZ patients, graph theory was used to analyze the dynamic ASSR activity. In general, the brain functional connectivity is regarded as a complex network with a large number of short edges and a small number of long edges. The threshold *T* was set to filter out the weak connections (those with small correlation values). The functional connectivity patterns were thus converted into a graph. It is important to note that threshold *T* is selected based on previous experience because there is no systematic method for its selection. Parameters *Cp* and *Lp* were computed at different thresholds. The results are shown in Fig. 4.

## Discussion

### Comparison of PSDA Mapping and Brain Connectivity

EEG signals measured with 32 channels were used in this study. The PSDA mapping in Fig. 3 shows that the ASSR driven by the 40-Hz stimulus mainly occurred in the central regions. While different from other SSEPs like SSVEP, the active regions were wide, even for the SZ patients. This may be due to the bilateral neurons consisting of the generators responsible for auditory oscillations. As shown in Fig. 3, long connections from the central to frontal and temporal regions were observed. Compared with PSDA results, which reflect the activation of only a single region, the functional connectivity pattern can show the process of global information transmission. The physiological areas related to auditory response were thus located and studied.

### Central Connection Cortex of ASSR

As shown in Fig. 3, the source of most activities was in the central regions. Yan and Gao [6] found that for SSVEP, the connection cortex is mainly located in the parietal regions. They proposed that the parietal regions act as a hub of brain information interchange, producing an output that reflects visual selection and preparation during the SSVEP task. The results of the ASSR suggest that the functional regions are also related to auditory stimulus. When a specific frequency stimulus is received, powerful activities and connections were found in the central regions, indicating brain resonation. Moreover, these connections were less involved in information interchange between other brain regions. It is possible that other sources in the central regions beyond the primary auditory cortex contribute to the ASSR [21, 22]. For the patients, an asymmetrical pattern between functional connectivity in left and right hemispheres was induced due to the abnormal function caused by auditory hallucinations.

### Structures of Network During ASSR

As shown in Fig. 4a, *Cp* is close to 1 for a low value of *T* (for *T* = 0, *Cp* is expected to be 1). If the value of *T* is too low, the corresponding graph will have full connectivity with edges between almost all nodes. As *T* is increased, more edges are lost, resulting in a decrease of *Cp*. In contrast, *Lp* increases with increasing *T*, so that more edges are lost when *T* is increased. When *T* > 0.02, the value of *Lp* started to drop off because some edges below the value of *T* were regarded as disconnections. The resulting connections in the graph thus became less common and the growth of *Lp* was limited.

In Fig. 4a, no remarkable differences can be observed between the normal subjects and SZ patients. However, in Fig. 4b, the average path length for the SZ patients is longer than that for the normal subjects between and across cortical regions. It has been shown that a short path length promotes effective interactions [23]. The interactions of interconnected brain regions are believed to establish the base mechanism of cognitive processes [7, 24]. The results of this study provide further evidence that brain function during auditory hallucinations was syndrome with disconnection. For auditory hallucinations, some defective neuronal connections decrease the connection speed between neurons, so information transfer during cognitive processes becomes inefficient.

## Conclusion

This study investigated the functional connectivity of the ASSR and the structure of the network of SZ patient with auditory hallucinations. The results indicate that the central region is an important neural location of human audio perception. An unordered network was found for SZ patients, suggesting that auditory hallucination syndrome could lead to a dysfunctional brain connectivity network. Preliminary studies of the network structures by graphical analysis were also conducted. The results show that patients with auditory hallucinations have disconnections and display the characteristics of small-world network loss. Graphical analysis was shown to be a useful approach for exploring the complexity patterns in brain networks.
